# Federated learning as a catalyst for digital healthcare innovations

**DOI:** 10.1016/j.patter.2024.101026

**Published:** 2024-07-12

**Authors:** Guang Yang, Brandon Edwards, Spyridon Bakas, Qi Dou, Daguang Xu, Xiaoxiao Li, Wanying Wang

**Affiliations:** Bioengineering Department and Imperial-X, Imperial College London, London W12 7SL, UK; National Heart and Lung Institute, Imperial College London, London SW7 2AZ, UK; Cardiovascular Research Centre, Royal Brompton Hospital, London SW3 6NP, UK; School of Biomedical Engineering & Imaging Sciences, King's College London, London WC2R 2LS, UK; Intel Corporation, Santa Clara, CA, USA; Division of Computational Pathology, Department of Pathology & Laboratory Medicine, Indiana University School of Medicine, Indianapolis, IN, USA; Center for Federated Learning in Medicine, Indiana University, Indianapolis, IN, USA; Department of Biostatistics & Health Data Science, Indiana University School of Medicine, Indianapolis, IN, USA; Department of Radiology & Imaging Sciences, Indiana University School of Medicine, Indianapolis, IN, USA; Department of Neurological Surgery, Indiana University School of Medicine, Indianapolis, IN, USA; Department of Computer Science, Luddy School of Informatics, Computing, and Engineering, Indiana University, Indianapolis, IN, USA; Medical Working Group, MLCommons, San Fransisco, CA, USA; Department of Computer Science and Engineering, The Chinese University of Hong Kong, Hong Kong, China; NVIDIA, Santa Clara, CA, USA; Electrical and Computer Engineering Department, The University of British Columbia, Vancouver, BC V6T 1Z4, Canada; Vector Institute, Toronto, ON M5G 0C6, Canada; Scientific editor, *Patterns*

## Main text

As the landscape of digital healthcare continues to evolve, the integration of artificial intelligence (AI) presents both immense opportunities and profound challenges. At the heart of this dynamic field lies the quest for innovative solutions that enhance patient care while safeguarding sensitive medical data. In response to these imperatives, the emergence of federated learning (FL) represents a pivotal advancement, offering a pathway to harness the collective intelligence of distributed healthcare datasets while respecting privacy and security protocols.

This special collection on “federated learning in digital healthcare” curated for *Patterns* stands as a testament to the growing significance of FL in revolutionizing healthcare AI. In an era where data is hailed as the new currency of innovation, FL emerges as a beacon of promise, addressing the twin pillars of data accessibility and privacy preservation. Central to the ethos of FL is its ability to transcend traditional data silos, enabling collaborative model training across diverse healthcare institutions without necessitating the sharing of raw patient data. This decentralized approach not only fosters a spirit of cooperation but also empowers organizations to leverage the collective wisdom inherent in their datasets, thereby fuelling advancements in medical research and clinical practice.

The articles featured in this special collection encapsulate a diverse array of perspectives and insights, ranging from theoretical frameworks to practical implementations. Through nine original research articles and three comprehensive reviews, thought leaders from academia and industry illuminate the transformative potential of FL in addressing the complexities of modern healthcare.

The research articles in this collection feature advanced and innovative approaches demonstrating various applications of FL in digital healthcare. These studies show that FL has been successfully implemented across diverse fields in healthcare. For example, Danek et al. explore the feasibility of FL in Parkinson’s disease prediction, while Rootes-Murdy et al. highlight the applications of FL in the detection of neurological and psychiatric disorders. Additionally, Yan et al. introduce an FL platform, FedEYE, for ophthalmologists to carry out machine learning (ML) research. The studies by Casella et al., Jiang et al., Danek et al., and Khalil et al. show that FL also benefits multimodal-data-type sharing across sites, including images, tabular data, multi-omics data, and textual data from social media. Furthermore, Pezoulas et al., Pan et al., and Zhang et al. offer solutions to challenges in the federated environment, such as imbalanced datasets, data distribution drifts, and unfairness in different data subgroups across different sites.

A series of reviews in this collection provides an in-depth understanding of the advantages and applications of FL in healthcare. In the review by Rajendran et al., they explain how FL is adopted to handle the challenges in data privacy in healthcare. From a methodology perspective, Zhang et al. provide a comprehensive literature review of the advanced methods since 2015, when FL was first introduced. The third review, by Pati et al. and co-authored by some of the authors of this Editorial, critically discusses the privacy threats during FL and associated mitigation techniques.

This collection underscores the profound implications of FL for patient-centric care, emphasizing the pivotal role of individuals in determining the trajectory of their healthcare journey. As we navigate the complexities of digital healthcare in the 21^st^ century, the insights gleaned from this special collection serve as a compass guiding us towards a future where innovation converges with ethics and technology becomes a catalyst for equitable and inclusive healthcare delivery. In embracing the principles of federated learning, we embark on a journey towards a more resilient, responsive, and patient-centric healthcare ecosystem.

